# The Role of Mitochondria-Derived Peptides in Cardiovascular Diseases and Their Potential as Therapeutic Targets

**DOI:** 10.3390/ijms22168770

**Published:** 2021-08-16

**Authors:** Siarhei A. Dabravolski, Nikita G. Nikiforov, Antonina V. Starodubova, Tatyana V. Popkova, Alexander N. Orekhov

**Affiliations:** 1 Department of Clinical Diagnostics, Vitebsk State Academy of Veterinary Medicine [UO VGAVM], 7/11 Dovatora Str., 210026 Vitebsk, Belarus; 2 Laboratory of Cellular and Molecular Pathology of Cardiovascular System, Institute of Human Morphology, 3 Tsyurupa Street, 117418 Moscow, Russia; nikiforov.mipt@googlemail.com (N.G.N.); a.h.opexob@gmail.com (A.N.O.); 3 Laboratory of Angiopathology, The Institute of General Pathology and Pathophysiology, 8 Baltiyskaya Street, 125315 Moscow, Russia; 4 Federal Research Centre for Nutrition, Biotechnology and Food Safety, 2/14 Ustinsky Passage, 109240 Moscow, Russia; avs.ion@yandex.ru; 5 Therapy Faculty, Pirogov Russian National Research Medical University, 1 Ostrovitianov Street, 117997 Moscow, Russia; 6 V.A. Nasonova Institute of Rheumatology, 34A Kashirskoye Shosse, 115522 Moscow, Russia; popkovatv@mail.ru

**Keywords:** mitochondria-derived peptides, cardiovascular diseases, Humanin, MOTS-c, SHLPs, atherosclerosis, insulin resistance, hyperlipidaemia, ageing

## Abstract

Mitochondria-derived peptides (MDPs) are small peptides hidden in the mitochondrial DNA, maintaining mitochondrial function and protecting cells under different stresses. Currently, three types of MDPs have been identified: Humanin, MOTS-c and SHLP1-6. MDPs have demonstrated anti-apoptotic and anti-inflammatory activities, reactive oxygen species and oxidative stress-protecting properties both in vitro and in vivo. Recent research suggests that MDPs have a significant cardioprotective role, affecting CVDs (cardiovascular diseases) development and progression. CVDs are the leading cause of death globally; this term combines disorders of the blood vessels and heart. In this review, we focus on the recent progress in understanding the relationships between MDPs and the main cardiovascular risk factors (atherosclerosis, insulin resistance, hyperlipidaemia and ageing). We also will discuss the therapeutic application of MDPs, modified and synthetic MDPs, and their potential as novel biomarkers and therapeutic targets.

## 1. Introduction

MDPs are a class of recently identified peptides, which are found within other known mitochondrial genes and encoded by small ORFs (open reading frames). The first MDP, HN (Humanin), was discovered in 2001 in patients with Alzheimer’s disease and described as a neuroprotective peptide with a high therapeutic potential for neurodegenerative diseases [1,2]. After HN, two other types of MDPs were discovered: MOTS-c (mitochondrial ORF of the 12S rDNA type-c) [3] and SHLP (small Humanin-like peptide, 1 to 6) [4]. MDPs are widely presented in different tissues, such as the kidney, skeletal muscle, colon, vascular wall and heart. MDPs are released into the body via paracrine and endocrine pathways and have diverse functions as cytoprotective agents, such as maintaining cell viability and mitochondrial function under stress, are involved in cellular metabolism and cell survival and act in response to inflammation and OS (oxidative stress) [5]. Recently, the role of MDPs was highlighted for many senescence and ageing-associated diseases, chronic inflammation diseases, cancer and neurodegenerative diseases and retinal and fertility diseases (reviewed in [6,7,8,9,10]). In this review, we focus on the role of on MDPs as crucial peptides, modulating and regulating mitochondrial function and involved in pathological changes in CVD via different molecular mechanisms. We also discuss the application of MDPs, modified MDPs and synthetic MDPs as uprising pharmaceutical tools for the treatment of CVD and other diseases. Further understanding the role of MDPs in various signalling pathways related to CVD would improve its medical significance and therapeutic potential.

## 2. MDP General Description

### 2.1. Mitochondrial Genome Overview

Mitochondria are complex organelles of bacterial origin, playing important roles in cellular signalling, energy production and metabolism. Mitochondria are known as partially autonomous organelles with their own genome, responsible for the synthesis of 4 out of 5 enzyme complexes of OXPHOS (oxidative phosphorylation) (complexes I, III, IV and V), 2 rRNAs (12S (small) and 16S (large)) and 22 tRNA (transfer RNA) to carry out intramitochondrial protein synthesis. MtDNA is compactly packed with only a regulatory D-loop non-coding region, which contains replication initiation sites and H-strand transcription promotors [11]. Many nuclear-encoded proteins are transported into mitochondria and required for proper functioning; however, some proteins are targeted back to the nucleus (so-called retrograde signalling) to influence nuclear gene expression [12]. Recently, researchers have found in the rRNA locus hidden ORFs that could be transcribed and translated into short peptides with outstanding biological properties [5]. Here, we will provide the known functions of these MDPs.

### 2.2. MDPs Generation and Functions

HN was the first identified MDP, encoded by an ORF in the 16S rRNA of mtDNA and translated into the 21 (in mitochondria) or 24-amino-acid peptide (in the cytoplasm). Despite the different lengths, 21 and 24-amino-acid peptides have similar biological functions. This polypeptide is conserved across species and involved in cell protection, anti-apoptosis, anti-inflammation and anti-oxidation, regulating mitochondrial biogenesis and functions [13,14,15], and activation of chaperon-mediated autophagy (via interaction with HSP90 (heat shock protein 90)) [13]. Wider investigation of different genomes provided data that the Humanin genes in many species are under pseudogenization but not in humans [16]. Furthermore, closer examination of the genomes of mice suggests that some nuclear genes could produce different Humanin-like peptides in different tissues and in different developmental stages and be involved in the regulation of mitochondria biogenesis [17]. The avian mitochondrial genome also contains conserved homologues of humanin and SHLPs, and, additionally, two new sORF (small open reading frames) called av-scO1 and av-scO2 (avian strongly conserved sORFs 1 and 2), encoding 17 and 25 amino-acid peptides, respectively [18]. Similarly, it was suggested that the human mitochondrial genome may carry more MDPs and some Humanin-like peptides are located inside many nuclear genes [19].

MOTS-c is the second identified 16 amino-acid MDP encoded by mtORF in the 12S rRNA gene. The best-known function of MOTS-c is based on the activation of AMPK (AMP-activated protein kinase); thus, MOTS-c regulates energy metabolism that could ameliorate IR (insulin resistance), diabetes, diet-induced obesity and other similar diseases [3]. Additionally, MOTS-c was shown to protect against coronary endothelial dysfunction by reduction of the release of several pro-inflammatory cytokines (TNF-α (Tumor Necrosis Factor-Alpha), IL-6 (Interleukin), IL-1β), adhesion molecules E-selectin, VCAM-1 (Vascular Cell Adhesion Molecule 1), ICAM-1 (Intercellular Adhesion Molecule) and inducible enzymes (COX2 (Cyclooxygenase-2), iNOS (Nitric Oxide Synthase 2A (Inducible, Hepatocytes))), thus inhibiting the MAPK/NF-κB pathway (mitogen-activated protein kinase/nuclear factor kappa-light-chain-enhancer of activated B cells) [20]. Interestingly, MOTS-c was also described as a specific retrograde regulator of nuclear gene expression via its interaction with several transcription factors [21]. Such MOTS-c activity was detected as a response to metabolic stress and, possibly, may be involved in the development of age-related diseases and regulation of human longevity [22].

SHLP1-6 (small Humanin-like peptides) were discovered in the same 16S rRNA as Humanin. While encoded by the same ORFs, they all have different biological functions. Among them, SHLP2 and SHLP3 are functionally the most similar to HN, with maximal expression in the spleen, kidney and liver, known as cytoprotective peptides [4]. SHLP1 was detected in the heart, SHLP2 in muscles and SHLP3 in the brain. The circulating level of SHLP2 in the blood is age-dependent, suggesting its role in the progression of age-related diseases and longevity. Recently, it was found that SHLP2 has chaperone-like properties, could increase the amounts of pancreatic cells and improve mitochondrial bioenergetics [23]. SHLP2 could also protect from age-related degeneration of macular cells via improved mitochondria functions and reduced apoptosis [24].

### 2.3. Nuclear-Encoded sORF Microproteins That Act on Mitochondria

Recently, several micropeptides acting on mitochondria were identified in nuclear sORFs. Thereby, a 54-amino-acid nuclear-encoded micropeptide called PIGBOS was shown to localize to the MOM (mitochondrial outer membrane) at the ER–mitochondria contact sites. PIGBOS interacts with the ER protein CLCC1 (Chloride Channel CLIC Like 1) and regulates UPR (unfolded protein response), and thus could be involved in the pathogenesis of many neurodegenerative disorders [25]. Another nuclear sORF-encoded micropeptide, MIEF1-MP (mitochondrial elongation factor 1 microprotein), was shown to localize to the mitochondrial matrix, interact with the mitochondrial ribosome and regulate the translation rate of the mitochondrial proteins [26]. Mitoregulin (also known as MPM (micropeptide in mitochondria) and MOXI (micropeptide regulator of β-oxidation)), a 56-amino-acid micropeptide encoded by long non-coding RNA (LINC00116), localizes to the MIM (mitochondrial inner membrane), where it binds cardiolipin and regulates Ca^2+^ metabolism, mitochondrial membrane potential, protein complex assembly and respiration rates [27]. Other functions of mitoregulin were defined in muscle cells, where it increases oxygen consumption, ATP production of mitochondria and could promote myogenic differentiation and muscle fibre growth [28] and fatty acid β-oxidation [29].

Interestingly, two Humanin-like nuclear genes (*MTRNR2L2* and *MTRNR2L8*) are upregulated and activate a “cell survival” network in patients with CAD (coronary artery disease) after 60 min of hyperoxia [30]. However, analysis of SNPs (single nucleotide polymorphisms) in 13 gene regions of Humanin-like peptides have found no statistically significant associations with CAD [31]. SNPs in Humanin-like nuclear isoform genes could influence CAD development on other molecular (post-transcriptional or post-translational), temporal (initial or more advanced disease progression stage) or genetic (specific ethnic group or mitochondrial DNA haplogroup) levels; thus, further research is required to specify their role in heart cell metabolism and heart diseases development.

Hopefully, with further technological improvements in the area of mitochondrial genome sequencing and small proteins identification, more MDPs and nuclear sORF-encoded micropeptides will be characterized and used as biomarkers or therapeutic targets.

### 2.4. Connection to Cardiovascular Risk Factors

Atherosclerosis, ageing, IR and hyperlipidaemia are the main risk factors for CVDs. As many researchers have shown, MDPs are closely associated with those risk factors.

Atherosclerosis: Atherosclerosis is a decades-lasting chronic inflammatory disease, which leads to the formation of foam cells, cholesterol accumulation in the arterial wall and is closely associated with CAD [32]. The foam cell formation is the initial stage of plaque development, which is based on the disbalance of the cholesterol influx and efflux in macrophages. Several receptors and transporters are involved in cholesterol transport and accumulation: CD36 (Fatty Acid Translocase), LOX-1 (Lectin-Type Oxidized LDL Receptor 1), ABCA1 (ATP-Binding Cassette Transporter A1) and ABCG1 (ATP-Binding Cassette Sub-Family G Member 1) [33]. Modified Humanin HNG (Gly [14]-Humanin with an amino-acid substitution at position 14 (Gly for Ser)) was shown to reduce the accumulation of Ox-LDL (Oxidized Low-Density Lipoprotein) by preventing *CD36* and *LOX-1* upregulation, and upregulate the expression of ABCA1 and ABCG1, thus preventing the formation of macrophage-derived foam cells [34]. Similarly, recent research has shown that HNG could directly suppress *LOX-1* expression [35]. Other research defines that HNG also promotes autophagic degradation of Ox-LDL throughout lysosomal enzyme cathepsin D and its membrane protein receptor FPRL1 (Formyl Peptide Receptor-Like 1) [36].

Hyperglycaemia-induced endothelial dysfunction plays a key role in the development of diabetes-associated premature atherosclerosis and further cardiovascular complications. Under high glucose conditions, vascular endothelial cells produce high levels of proinflammatory cytokines, such as TNF-α and IL-1β, ROS (reactive oxygen species) and high levels of VCAM-1 and E-selectin, which initiate atherosclerosis via adhesion of circulating leukocytes to the endothelium [37]. The reduced expression level of *KLF2* (Krüppel-like factor 2), an essential regulator of endothelial functions, also was found to be closely related to hyperglycaemia-induced endothelial dysfunction. KLF2 protects endothelial cells via regulation of the angiogenesis, thrombotic activation and endothelial pro-inflammatory pathway [38]. As it was recently shown, Humanin treatment induces the expression of *KLF2* and regulates the expression of the *KLF2* target genes, such as *eNOS* (endothelial nitric oxide synthase) and *ET-1* (endothelin-1). Humanin treatment also inhibits the high glucose-induced secretion of TNF-α and IL-1β and reduces the expression of *VCAM-1* and *E-selectin*, thus preventing hyperglycaemia-induced attachment of the monocytes to the vascular cells, endothelial dysfunction and atherosclerosis progression [39]. 

Similar effects were shown for MOTS-c treatment, which decreased the expression levels of the *AT-1* (angiotensin II type 1) and *ET-B* (endothelin B) receptors and increased the level of phosphorylated AMPK [40]. AT-1 is a crucial player in CVD and an important drug target; the high level of the AT-1 receptor leads to myocardial fibrosis, cardiac dysfunction and heart failure, while a decreased level of the AT-1 receptor is associated with reduced oxidative stress, thus preventing the cardiac remodelling and development of myocardial contractile dysfunction [41]. Similarly, the MOTS-c level correlates with microvascular and epicardial endothelial function, and thus could be used as a marker for early coronary atherosclerosis [42] and CAD [43].

Ageing: In humans, plasma levels of MDPs (HN, MOTS-c and SHLP2) decline with age and correlate with mitochondrial dysfunction, rise in mitochondria-generated oxidative damage and development of age-related diseases (reviewed in [44,45]).

Cellular senescence is usually connected with the production of SASPs (senescence-associated secretory phenotypes), which are required to attract immune cells to remove senescent cells. Administration of HN and MOTS-c to the senescent cells increases secretion of some SASP components and raises mitochondrial respiration, suggesting their cytoprotective role in mitochondria energy metabolism and therapeutic potential for the lifespan extension [46]. Similarly, MOTS-c and HN could stimulate the secretion of some SASP components (cytokines IL-1β, IL-6, IL-8, IL-10 and TNF-α) and make SASP-secreting senescent cells easier to detect and cleared by immune cells [8]. Recent research has shown that levels of HN, cytoprotective factor GDF15 (Growth Differentiation Factor 15) and major metabolic regulator FGF21 (Fibroblast Growth Factor 21) positively correlates with age in humans and could be considered as biomarkers of biological age [47] and age-associated diseases with known mitochondrial impairment [48].

Insulin resistance: Insulin resistance in the metabolic tissues is often associated with a decreased number of mitochondria and available oxidative enzymes, abnormal mitochondria morphology, reduced production of ATP and insulin-dependent glucose disposal [49,50]. Humanin administration was shown to increase insulin sensitivity in muscles and the liver, fatty acid metabolism signalling and insulin-mediated AKT-signalling (Protein kinase B) [51,52]. Treatment of Alzheimer’s disease in a mice model (APP/PS1 transgenic mice, which is expressing a chimeric mouse/human amyloid precursor protein (Mo/HuAPP695swe) and a mutant human presenilin 1 (PS1-dE9), both directed at the central nervous system neurons) with HNG improved insulin sensitivity in the brain through the regulation of the IRS-1/mTOR (Insulin Receptor Substrate 1/mechanistic target of rapamycin) insulin signalling pathway in the hippocampus [53]. Similar to HN, SHLP2 and SHLP3 also have confirmed in vivo and in vitro insulin-sensitizing activities. The primary activity of SHLP2 and SHLP3 has been shown on adipose tissue, where they enhanced 3T3-L1 pre-adipocyte differentiation. Infused SHLP2 also increased the glucose uptake and suppressed hepatic glucose production, thus acting as both a central and peripheral insulin sensitizer [4].

MOTS-c promotes system-level insulin sensitivity via skeletal muscle, where it increases fatty acids β-oxidation and mitochondria biogenesis. Mechanically, MOTS-c acts throughout the AMPK, SIRT1 (Sirtuin 1) and PGC1α (Peroxisome Proliferator-Activated Receptor Gamma Coactivator 1-Alpha) signalling pathways, and stimulates *GLUT4* (Glucose Transporter Type 4, Insulin-Responsive) expression [54,55].

Hyperlipidaemia: Humanin is also one of the central regulators of peripheral lipid metabolism. HN treatment of human hepatocytes resulted in decreased lipid accumulation and downregulation of lipogenesis genes (*SREBP1* (Sterol Regulatory Element Binding Transcription Factor 1), *FAS* (Fatty Acid Synthase), and *SCD1* (Acyl-CoA Desaturase)). In addition to an enhanced AMPK phosphorylation-mediated effect on IR, HN also suppressed phosphorylation of the mTOR signalling pathway [56], the crucial regulator of response to stresses, protein synthesis, cell growth and proliferation, cell survival and cell cycle progression [57]. Injection of HNG was shown to decrease liver triglyceride accumulation, visceral fat and body weight gain in high-fat diet-fed mice [58]. Similarly, injections of HNG and SHLP2 in a diet-induced obesity mouse altered the concentrations of amino acid and lipid metabolites in plasma, acting mostly via the glutathione and sphingolipid metabolism pathways [59].

MOTS-c, acting mostly via skeletal muscle, targets the methionine-folate cycle and connected de novo purine biosynthesis, increases the AICAR (5-aminoimidazole-4-carboxamide ribonucleotide) levels and activates AMPK, thus ameliorating IR and diet-induced obesity [3]. On the molecular level, PGC-1α regulates the involvement of MOTS-c in energy metabolism, where further MOTS-c activates AMPK, GLUT4 and ACC (Acetyl CoA carboxylase), stimulating mitochondria biogenesis and increasing the level of fatty acid β-oxidation [60]. In the D-galactose fed mice, MOTS-c treatment was shown to alleviate the lipid accumulation through improved mitochondria dynamics (achieved via altering mRNA levels of Drp1 (Dynamin-Related Protein 1) and mitofusins) [61]. Taking into account the ability of MOTS-c under different metabolic stresses to translocate to the nucleus and regulate the expression of many genes, primarily with *ARE* (antioxidant response elements) and TF (such as *NFE2L2*/*NRF2* (nuclear factor erythroid 2-related factor 2)) [21], it could be considered as a crucial player in the metabolism of glycolipids and a promising target for the treatment of metabolic diseases.

Discussed connections between MDPs and CVD risk factors are summarized in Table 1.

### 2.5. Mechanisms of Action

The main therapeutic properties of MDPs are anti-inflammation and anti-apoptosis, offering protection from OS and ER stress. In the following, we briefly discuss the molecular mechanism of these pathways.

Anti-inflammatory mechanism: The anti-inflammatory effect of MOTS-c treatment relies on inhibition of pro-inflammatory cytokines (IL-1β, IL-6 and TNFα) through decreased phosphorylation of MAPK and upregulating the levels of TF STAT3 (signal transducer and activator of transcriptional 3) and AhR (aryl hydrocarbon receptor) in macrophages [3,62]. STAT3 and AhR are involved in many cellular biological processes via the regulation of anti/proinflammatory responses [63,64]. Similarly, the HN treatment resulted in the inhibition of the expression of *IL-1β* and *IL-18* and prevented AMPK-mediated NLRP3 (NOD-, LRR- and pyrin domain-containing protein 3) inflammasome activation [65]. The NLRP3 inflammasome plays a central role in the pathophysiology of atherosclerosis and CAD development [66,67]. On the contrary, SHLP3 treatment increased the levels of IL-6 and MCP-1 (monocyte chemotactic protein-1), while SHLP2 treatment had no such effect [4].

Protection from OS and ER stress: Mitochondria are the main energy-producing organelles, but also the main ROS producer, which causes oxidative damage to proteins, lipids and mtDNA, and is involved in the development of low-grade chronic inflammation, ageing, CVD and premature death [68,69]. However, experiments on several cell lines (such as retinal pigment epithelium, lens epithelial cells, cortical neurons and neuroblastoma cancer cells) proved that HN could protect mitochondria and cells from oxidative stress and ER stress [70,71,72,73]. HN acts via a mitochondrial antioxidant defence system, where HN stimulates expression of *SOD1* (Superoxide Dismutase 1), biosynthesis and restoring the mitochondrial pool of glutathione [72,74]. Both factors (glutathione and SOD1) are known cardioprotectors against ischemia–reperfusion injury and other CVDs [75]. Other research has shown that HN treatment protects retina cells from OS-induced cell death by ameliorating mitochondrial functions (upregulated mtTFA (Mitochondrial transcription factor A) and increased mtDNA copy number), stimulate STAT3 phosphorylation and inhibit caspase-3 activation [76]. The ability of HN to inhibit the ROS-dependent JNK/p38 MAPK (Janus Kinase/Mitogen-Activated Protein Kinase 14 or P38 MAP Kinase) signalling pathway was also shown in a cortical neurons model [77]. In experiments on human aortic endothelial cells, HN treatment protects from OS via downregulation of *NOX2* (Superoxide-Generating NADPH Oxidase Heavy Chain Subunit) and *TxNIP* (Thioredoxin Interacting Protein) gene expression and leads to a reduction in ROS and protein carbonyl [65]. *NOX2* is the main player in cardiac oxidative damage [78], while *TxNIP* and protein carbonyl are markers of metabolic and oxidative stress [79,80]. Similarly, protective properties against oxidative stress were shown for HN analogue HNG on SH-SY5Y neuroblastoma cells, where HNG acted via the PI3K/AKT (phosphatidylinositol 3-kinase/protein kinase B) pathway [81], a crucial signalling pathway involved in the regulation of apoptosis, cell differentiation and proliferation [82]. Further details of the HN-mediated protection against OS in CVD could be found in a recent review [83].

Anti-apoptosis properties: Humanin plays an important role in cytoprotection as an anti-apoptosis factor. Several studies have shown that HN is involved in the interaction with several signalling pathways improving cell survival and preventing cell death: (1) plasma membrane receptors FPRL1 and FPRL2; (2) cytokine-like receptors CNTF (Ciliary Neuronotrophic Factor), IL27RA (Interleukin 27 Receptor Subunit Alpha) and IL6ST (Interleukin 6 Cytokine Family Signal Transducer) to affect the JAK and STAT3 signalling pathways; (3) MAPK14 (Mitogen-Activated Protein Kinase 14) and MAPK3 (Mitogen-Activated Protein Kinase 3); (4) AMPK; (5) BAX (BCL2 Associated X, Apoptosis Regulator) and BCL2L11 (BCL2-Like 11 Apoptosis Facilitator); (6) IGFBP-3 (insulin-like growth factor-binding protein 3) reviewed in [84]; and (7) inhibiting the membrane association and oligomerization of the apoptosis regulators and mediators of mitochondrial damage Bax and Bid (BH3-Interacting Domain Death Agonist) proteins [85,86,87]. Similarly, anti-apoptosis properties were shown for SHLP2 and SHLP3, while SHLP6 increased apoptosis [4]. An anti-apoptosis effect was also shown for the Humanin analogue HNG, which was checked on chondrocytes of arthritis and severe combined immunodeficiency mice models [88,89].

However, opposite properties were defined for HN in cancer cells. It was defined that *HN* was upregulated in TNBC (triple-negative breast cancer) model biopsies. Application of HN in a TNBC mice model leads to a reduction in the tumour apoptotic rate, stimulates tumour progression, growth and lung metastases development, thus abating chemotherapy effects [90]. Thereby, HN may have tumour-stimulation activity and a tumour HN-blocking strategy could be considered as a possible target to increase the efficacy of chemotherapy in breast cancer treatment.

**Figure 1:**
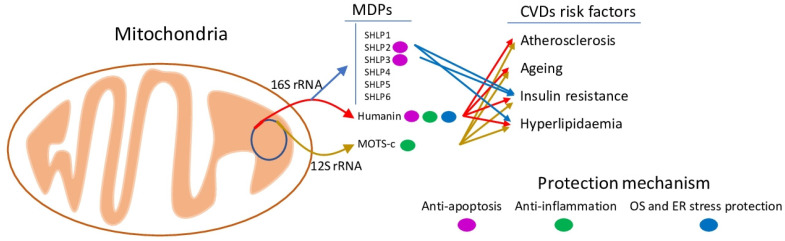
Classification and protection mechanisms of MDPs against CVD risk factors. MDPs (Humanin, SHLPs and MOTS-c) are hidden in mitochondrial 16S and 12S rRNA genes. MDPs are associated with CVD risk factors (atherosclerosis, ageing, insulin resistance and hyperlipidaemia). The main cardioprotective activities identified for MDPs are anti-apoptosis and anti-inflammation, offering protection from OS and ER stresses.

In sum, we can conclude that MDPs have a strong connection with the main CVD risk factors (atherosclerosis, hyperlipidaemia, insulin resistance and ageing) and act as anti-apoptosis and anti-inflammation agents, providing protection against ROS and OS (Figure 1). However, current research data are mostly obtained on HN and its modification, HNG, while the bioactive properties of SHLPs and MOTS-c are less studied and require more attention. The potential tumour-stimulating properties of MDPs and the effect of MDP-silencing tools on the efficacy of chemotherapy should be further studied.

## 3. Association between MDPs and CVDs

Coronary microvascular dysfunction: CMD (coronary microvascular dysfunction) refers to functional and structural abnormalities in small coronary vessels. CMD carriers have an increased risk of the development of hypertensive heart disease, heart failure, chronic inflammatory, autoimmune diseases and diabetes; also, it was shown that inflammation and ED (endothelial dysfunction) are the main cause of CMD [91]. The HN level is decreased in CMD patients [92], thus suggesting that the CMD protective properties of HN are based on anti-inflammation and anti-atherosclerosis activities on ED. These results also suggest HN as a biomarker of ED and a possible therapeutic target for CMD treatment. Similarly, decreased levels of MOTS-c correlate with ED [42]. However, the exact molecular mechanisms of MDPs on CMD are not known and require further investigation.

Myocardial fibrosis: Myocardial fibrosis refers to histological changes in the myocardium through increased myofibroblast activity and excessive extracellular matrix deposition, identified in several chronic cardiac diseases and eventually leading to heart failure and death [93]. The cardioprotective effect of HNG treatment on myocardial fibrosis and apoptosis was shown on aged mice, where HNG application significantly reduced apoptosis, collagen deposition in aged hearts, cardiac fibroblast proliferation and expression of *MMP2* (Matrix Metallopeptidase 2), *FGF2* (Fibroblast Growth Factor 2) and *TGFB1* (Transforming Growth Factor Beta 1). Mechanically, HNG acts via upregulation of the *AKT*/*GSK3B* (Protein kinase B/Glycogen Synthase Kinase 3β) pathway [94]. Elevated expression of *FGF2* and *MMP2* in the ageing heart is associated with the promotion of cardiac fibrosis [95]. Mechanically, pGSK-3β relies on the amelioration of mitochondrial function, suppression of ER stress and myocardial apoptosis [96].

Myocardial ischemia and reperfusion injury: AMI (acute myocardial ischemia) is a leading cause of death worldwide. This sudden blockage of cardiac blood vessels leads to decreased or completely stopped blood flow to a part of the heart, causing heart damage or necrosis. PCA (percutaneous coronary angioplasty) treatment could quickly and effectively restore the blood flow in the damaged area and reduce the associated morbidity and complications. However, such blood flow restoration to the ischemic zone causes a significant rise in damaging ROS, called MRI (myocardial reperfusion injury), and is associated with further complications (reperfusion arrhythmias, myocardial stunning and lethal reperfusion) [97,98].

According to published research, MDPs protect the heart in AMI and MRI. Application of HNG during the ischemic period attenuated the heart mitochondrial dysfunction, resulting in increased HN levels in the damaged myocardium and decreased left ventricular dysfunction, myocardial infarct size and cardiac arrhythmia [99,100]. The OS and ROS cause cardiac mitochondria swelling, depolarization and reduced ATP production. These effects were attenuated by HNG treatment, which protects mitochondria through decreased complex I activity [101]. Similarly, HNG protects brain mitochondria during AMI and MRI, reducing tau hyperphosphorylation, Aβ accumulation and apoptosis [102,103]. However, in the porcine model system, the cardioprotective effect of HNG treatment was observed only for short-term ischemia, with no significant protective effect with prolonged ischemia [104]. In this research, the inhibition of apoptosis was suggested as a primarily protective mechanism. Recently, a novel role of HNG in platelet function and thrombus formation was defined [105]. HNG treatment inhibited platelet aggregation, P-selectin expression, αIIbβ3 activation and adhesion under flow conditions. The potential molecular mechanism of microtubule stabilization was suggested as enhanced tubulin acetylation and inhibited microtubule depolymerization [105].

In total, the presented data suggest that MDPs protect the heart and vessels from disease-causing threads by improving mitochondrial functioning, reducing the consequences of OS damage and elevated ROS levels. Anti-inflammation and anti-atherosclerosis effects protect against endothelial dysfunction, while the anti-apoptotic effect is a primary protective mechanism against myocardial ischemia and reperfusion injury.

The discussed connections between CVDs and MDPs are summarized in Table 2.

## 4. Therapeutic Application of Modified MDPs

Among all MDPs, HN is the first identified and better studied, with diverse biological activities in apoptosis, inflammation, cell stress responses and metabolism modification, thus making it an attractive candidate for application as a therapeutic agent. However, as a short peptide, HN is subjected to rapid tissue clearance, resulting in low availability. Similar to the neuroprotective effect of HN [2], the neuroprotective properties of the primary HN modification (HNG (S14G)) were investigated in the original report [1], which has a 1000-fold stronger biological activity than unmodified Humanin [81]. While the biological properties of HNG have been widely covered in this manuscript, we further focus on other HN modifications and their therapeutic application.

Recently, the neuroprotective and myoprotective effects were evaluated for the synthetic HN analogues HUJInin (resulted from the conjugation of HN modification (G14) HNG17-NH2 and NAP (an 8-amino-acid neuroprotective peptide NAPVSIPQ, also called davundetide) and cyclic(D-Ser14)AGA-(C8R)humanin [106]. The neuroprotective effect was dose-dependent and relies on improvement of the mitochondrial functions, stimulation of AKT phosphorylation and attenuation of insult-induced Erk1/2 (Mitogen-Activated Protein Kinase 1 and 3) phosphorylation. A myoprotective effect was observed towards doxorubicin-induced apoptosis and necrosis cell death insults [106]. Doxorubicin is a crucial anti-cancer and cytotoxic drug, which is used in different chemotherapy protocols for the treatment of lymphoma, breast cancer and leukaemia. However, cardiotoxicity is one of the main life-threatening side effects of doxorubicin treatment, resulting in significant morbidity and mortality in cancer patients [107]. Thus, further improvement of HUJInin and c(D-Ser14-HN) could lead to the development of new drugs to treat stroke and/or doxorubicin-induced cardiotoxicity in cancer patients. HNG was also effectively used in combination with dexrazoxane, an approved drug against dox-induced cardiotoxicity [108].

Strong neuroprotective properties were shown for the HN analogue called colivelin (AGA-(C8R)HNG17, attached to the C-terminus of ADNF (activity-dependent neurotrophic factor)).

Experiments on APPswe/PS1dE9 mice (Alzheimer’s disease model) suggested that colivelin treatment improve the cognitive and behavioural functions and reduced Aβ deposition in the hippocampus in APP/PS1 mice [109]. Colivelin was also shown to protect neurons after ischemic brain injury. In mice, subjected to 60 min induction of transient focal cerebral ischemia and reperfusion, colivelin administration activated the STAT3 signalling pathway, inhibited axonal damage and neuronal death in brain tissue, increased axonal growth and decreased the neurological deficits and infarct lesion induced by brain ischemia [110]. Thus, colivelin is a promising drug candidate that could be used as a single or adjunct therapy in Alzheimer’s disease and ischemic stroke.

The efficiency of recombinant fusion between HN and thermally responsive ELP (elastin-like polypeptides) against human AMD (age-related macular degeneration) was recently evaluated. Thereby, HN facilitates cellular delivery of biodegradable nanoparticles, binds human RPE (retinal pigment epithelium) cells and protects against OS-mediated apoptosis [70]. Similarly, the Humanin derivative AGA-HNG (AGA-(CR8)HNG17) encapsulated in chitosan nanoparticles was shown to reduce the inflammatory response characteristic of AMD modulated on ARPE-19 (immortalized retinal pigment epithelial) cells. Nano-encapsulated AGA-HNG significantly decreased apoptosis in hypoxic cells and reduced expression of *VEGF* (Vascular endothelial growth factor) with reduced cellular toxicity in comparison to the free drug [111]. Currently, anti-VEGF therapy is the most promising treatment in ophthalmology [112], and, because VEGF is produced and acts only locally, the application of nano-encapsulated HN analogues could provide a valuable drug candidate to reduce the pathogenesis characteristic of AMD with minimum toxic effects to cells.

The application of HN and its analogues in cancer treatment is a promising area to explore. As it was recently shown, silencing of the HN gene with BV-shHN (Baculovirus-encoded short-hairpin RNA) had a cytoprotective effect in pituitary tumour cells. Further, intratumor injection of BV-shHN into nude mice with pituitary adenoma increased the number of apoptotic cells, delayed tumour growth and enhanced their survival rate, suggesting that HN is involved in pituitary tumour growth and progression and could be a target for therapeutic intervention in the treatment of pituitary tumours [113]. HN also has an inhibitory effect on lung cancer cells growth. The molecular pathway relies on circNOL10 (circular RNA NOL10), which promotes the expression of TF *SCML1* (sex comb on midleg-like 1)—one of the regulators of HN expression [114]. Thus, circNOL10 could serve as a novel target for the molecular therapy of lung cancer with HN-mediated improvement of mitochondrial function, inhibition of tumour cell proliferation and cell cycle progression, thereby inhibiting lung cancer development.

Neuroprotection was the first discovered function of HN; however, the presented data suggest that HN has a wide area of application against other diseases, including different types of cancer. Detailed molecular mechanisms of action and activated pathways are mostly unknown and require further investigation. Recent progress allows to significantly increase the biologic activity of the synthetic HN analogues, packing it into nanoparticles for delivery to the required place of action and silencing HN in a tissue-specific way, thus extending the therapeutic application of HN.

## 5. Conclusions

Identified mitochondrial-derived peptides are hidden in mitochondria genes and closely associated with human mitochondria metabolism as well as neurodegenerative and age-related diseases. The main studied activities of MDPs are linked to cytoprotection, which is achieved via amelioration of mitochondrial functions by OS and ER stress protection, and anti-inflammation mechanisms. In this review, we summarized the recent progress in understanding the functions of MDPs in cardioprotection against the main CVD risk factors (atherosclerosis, hyperlipidaemia, ageing and insulin resistance). We could conclude that MDPs could be considered as promising targets in the treatment of CVD, protecting the heart and vascular endothelial cells. However, some MDPs have been assigned with undesirable activities (stimulate the production of pro-inflammatory cytokines, promote tumour growth and decrease the efficacy of chemotherapy); thus, further studies are required to eliminate these activities or minimize their influence. Recent research also suggests the existence of new MDPs encoded by mitochondrial genes and mitochondria-acting micropeptides encoded by the nuclear genome. Modern research techniques, applying nanotechnologies and in vitro peptide synthesis, allow to greatly increase biologic activity and deliver MDPs directly to the place of action, thus further expanding the therapeutic application of MDPs.

## Figures and Tables

**Table 1:** Identified connections between MDPs and CVD risk factors.

CVD Risk Factor	MDP	The Way of Action	References
Atherosclerosis	HNG	prevents *CD36* and *LOX-1* upregulation; upregulates the expression of *ABCA1* and *ABCG1*	[34]
		suppress *LOX-1* expression	[35]
		promotes autophagic degradation of Ox-LDL throughout lysosomal enzyme cathepsin D and its receptor FPRL1	[36]
	NH	induces the expression of *KLF2*;inhibits the high glucose-induced secretion of TNF-α and IL-1β; reduces the expression of *VCAM-1* and *E-selectin*	[39]
	MOTS-c	decreases the expression levels of the *AT-1* and *ET-B* receptors; increased the level of phosphorylated AMPK	[40]
Ageing	MOTS-c and HN	increase secretion of SASP components and rise mitochondrial respiration	[46]
		stimulate the secretion of SASP components (cytokines IL-1β, IL-6, IL-8, IL-10 and TNF-α)	[8]
Insulin resistance	HN	increases insulin sensitivity in muscles and the liver; increase fatty acid metabolism signalling and insulin-mediated AKT-signalling	[51,52]
	HNG	improves insulin sensitivity in the brain through the regulation of IRS-1/mTOR signalling pathway in the hippocampus	[53]
	SHLP2 and SHLP3	enhance 3T3-L1 pre-adipocyte differentiation	[4]
	SHLP2	SHLP2 increased glucose uptake and suppressed hepatic glucose production	[4]
	MOTS-c	increases fatty acids β-oxidation and mitochondria biogenesis; stimulates *GLUT4* expression	[54,55]
Hyperlipidaemia	HN	decreases lipid accumulation and down-regulates lipogenesis genes (*SREBP1*, *FAS* and *SCD1*); enhances AMPK phosphorylation-mediated effect on IR; suppresses phosphorylation of the mTOR signalling pathway	[56]
	HNG	decreases liver triglyceride accumulation, visceral fat and body weight gain in high-fat diet-fed mice	[58]
	HNG and SHLP2	acting via the glutathione and sphingolipid metabolism pathways alter the concentrations of amino acid and lipid metabolites in plasma	[59]
	MOTS-c	increases AICAR levels and activates AMPK	[3]
		activates AMPK, GLUT4 and AC; stimulating mitochondria biogenesis and increasing the level of fatty acid β-oxidation	[60]
		improves mitochondria dynamics	[61]
		regulates expression of ARE and NFE2L2/NRF2	[21]

**Table 2:** Identified connections between CVDs and MDPs.

CVD	MDP	The Way of Action	References
CMD	HN	HN level is decreased in CMD patients; HN is a biomarker of ED	[92]
	MOTS-c	MOTS-c level correlates with ED	[42]
Myocardial fibrosis	HNG	reduces apoptosis, collagen deposition in aged hearts, cardiac fibroblast proliferation and expression of *MMP2*, *FGF2* and *TGFB1;* up-regulates *AKT*/*GSK3B* pathway	[94]
Myocardial ischemia and reperfusion injury	HNG	attenuates the heart mitochondrial dysfunction; decreases left ventricular dysfunction, myocardial infarct size and cardiac arrhythmia	[99,100]
		attenuates cardiac mitochondria swelling, depolarization and reduced ATP production; decreases mitochondrial complex I activity.	[101]
		protects brain mitochondria during AMI and MRI, reducing tau hyperphosphorylation, Aβ accumulation and apoptosis	[102,103]
		inhibits platelet aggregation, *P-selectin* expression, αIIbβ3 activation and adhesion under flow conditions; enhances tubulin acetylation and inhibits microtubule depolymerization	[105]

## Data Availability

Not applicable.

## Abbreviations

ABCA1	ATP-binding cassette transporter A1
ABCG1	ATP-binding cassette sub-family G member 1
ACC	acetyl CoA carboxylase
ADNF	activity-dependent neurotrophic factor
AhR	aryl hydrocarbon receptor
AICAR	5-aminoimidazole-4-carboxamide ribonucleotide
AKT	protein kinase B
AMD	age-related macular degeneration
AMI	acute myocardial ischemia
AMPK	AMP-activated protein kinase
ARPE-19	immortalized retinal pigment epithelial
AT-1	angiotensin II type 1
av-scO1	avian strongly conserved sORFs 1
BAX	BCL2 Associated X, apoptosis regulator
BCL2L11	BCL2-like 11 apoptosis facilitator
Bid	BH3-interacting domain death agonist
BV-shHN	Baculovirus-encoded short-hairpin RNA
CAD	coronary artery disease
CD36	fatty acid translocase
CLCC1	chloride channel CLIC like 1
CMD	coronary microvascular dysfunction
CNTF	ciliary neuronotrophic factor
COX2	cyclooxygenase-2
CVD	cardiovascular disease
Drp1	dynamin-related protein 1
ED	endothelial dysfunction
ELP	elastin-like polypeptides
eNOS	endothelial nitric oxide synthase
Erk1/2	mitogen-activated protein kinase 1 and 3
ET-1	endothelin-1
ET-B	endothelin B
FAS	fatty acid synthase
FGF2	fibroblast growth factor 2
FGF21	fibroblast growth factor 21
FPRL1	formyl peptide receptor-like 1
GLUT4	glucose transporter type 4, insulin-responsive
GSK3B	glycogen synthase kinase 3β
HN	Humanin
HSP90	heat shock protein 90
ICAM-1	intercellular adhesion molecule
IGFBP-3	insulin-like growth factor-binding protein 3
IL-6	interleukin
IL27RA	interleukin 27 receptor subunit alpha
IL6ST	interleukin 6 cytokine family signal transducer
iNOS	nitric oxide synthase 2A inducible, hepatocytes
IR	insulin resistance
IRS-1	insulin receptor substrate 1
JNK	Janus kinase
KLF2	Krüppel-like factor 2
LOX-1	lectin-type oxidized LDL receptor 1
MAPK	mitogen-activated protein kinase
MAPK14	mitogen-activated protein kinase 14 or p38 MAP kinase
MAPK3	mitogen-activated protein kinase 3
MCP-1	monocyte chemotactic protein-1
MDPs	mitochondria-derived peptides
MIEF1-MP	mitochondrial elongation factor 1 microprotein
MIM	mitochondrial inner membrane
MMP2	matrix metallopeptidase 2
MOM	mitochondrial outer membrane
MOTS-c	mitochondrial ORF of the 12S rDNA type-c
MOXI	micropeptide regulator of β-oxidation
MPM	micropeptide in mitochondria
MRI	myocardial reperfusion injury
mTOR	mechanistic target of rapamycin
mtTFA	mitochondrial transcription factor A
NF-κB	nuclear factor kappa-light-chain-enhancer of activated B cells
NFE2L2	nuclear factor erythroid 2-related factor 2
NLRP3	NOD-, LRR- and pyrin domain-containing protein 3
NOX2	superoxide-generating NADPH oxidase heavy chain subunit
ORF	open reading frames
OS	oxidative stress
Ox-LDL	oxidized low-density lipoprotein
OXPHOS	oxidative phosphorylation
PCA	percutaneous coronary angioplasty
PGC1α	peroxisome proliferator-activated receptor gamma coactivator 1-alpha
PI3K	phosphatidylinositol 3-kinase
ROS	reactive oxygen species
RPE	retinal pigment epithelium
SASPs	senescence-associated secretory phenotypes
SCD1	acyl-CoA desaturase
SCML1	sex comb on midleg-like 1
SHLP	small humanin-like peptide, 1 to 6
SIRT1	Sirtuin 1
SNPs	single nucleotide polymorphisms
SOD1	superoxide dismutase 1
SREBP1	sterol regulatory element-binding transcription factor 1
STAT3	signal transducer and activator of transcriptional 3
TGFB1	transforming growth factor-beta 1
TNBC	triple-negative breast cancer
TNF-α	tumour necrosis factor-alpha
TxNIP	thioredoxin interacting protein
UPR	unfolded protein response
VCAM-1	vascular cell adhesion molecule 1
VEGF	vascular endothelial growth factor
